# VEGF Promotes the Transcription of the Human *PRL-3* Gene in HUVEC through Transcription Factor MEF2C

**DOI:** 10.1371/journal.pone.0027165

**Published:** 2011-11-02

**Authors:** Jianliang Xu, Shaoxian Cao, Lu Wang, Rui Xu, Gong Chen, Qiang Xu

**Affiliations:** State Key Laboratory of Pharmaceutical Biotechnology, School of Life Sciences, Nanjing University, Nanjing, China; University of Illinois at Chicago, United States of America

## Abstract

Phosphatase of regenerating liver 3 (PRL-3) is known to be overexpressed in many tumors, and its transcript level is high in the vasculature and endothelial cells of malignant tumor tissue. However, the mechanism(s) underlying its enhanced expression and its function in endothelial cells remain unknown. Here, we report that vascular endothelial growth factor (VEGF) can induce *PRL-3* transcription in human umbilical vein endothelial cells (HUVEC). An analysis of its 5′UTR revealed that *PRL-3* transcription is initiated from two distinct sites, which results in the formation of the two transcripts, *PRL-3-iso1* and *PRL-3-iso2*, but only the latter is up-regulated in HUVEC by VEGF. The *PRL-3-iso2* promoter region includes two functional MEF2 (myocyte enhancer factor2) binding sites. The over-expression of the constitutively active form of MEF2C promotes the abundance of the *PRL-3-iso2* transcript in a number of human cell lines. The siRNA-induced knockdown of MEF2C abolished the stimulative effect of VEGF on *PRL-3* transcript in HUVEC, indicating that the VEGF-induced promotion of *PRL-3* expression requires the presence of MEF2C. Finally, blocking PRL-3 activity or expression suppresses tube formation by HUVEC. We suggest that PRL-3 functions downstream of the VEGF/MEF2C pathway in endothelial cells and may play an important role in tumor angiogenesis.

## Introduction

PRL-3 is a member of the phosphatase of regenerating liver (PRL) family which represents a novel family of small (∼22 kDa) highly homologous protein tyrosine phosphatases (PTPs) [1]. Since the presence of PRL-3 was first linked with colon cancer metastasis in 2001 [2], evidence has accumulated that this protein is associated with various oncogenic and metastatic processes [3]. PRL-3 is abundant in many cancer cell lines and metastatic lesions, including gastric cancer [4], malignant melanoma cancer [5], ovarian cancer [6], breast cancer [7], colonic cancer [8] and esophageal squamous cell carcinoma [9]. For this reason, it is now commonly referred to as a metastasis-associated phosphatase [10], [11], [12], and its importance in cancer cell invasion and migration has been widely demonstrated [13], [14], [15].

Within a developing tumor, oxygen and nutrition are supplied via angiogenesis. VEGF is released into the extracelluar matrix by cancer cells to stimulate the migration of endothelial cells towards the source of the VEGF signal, where they proceed to form immature vessels via vasculogenesis or angiogenic sprouting [16], [17]. MEF2C, a member of the myocyte enhancer factor 2 (MEF2) family of transcription factors originally identified as activators of muscle differentiation [18], [19], appears to play a particularly important role in angiogenesis during vascular development. The deletion of MEF2C in mice results in embryonic lethality associated with significant cardiovascular defects, and the phenotype of MEF2C deficient mice is similar to that of mice lacking VEGF [20], [21], [22]. VEGF has also been reported to induce the expression of MEF2C and to stimulate MEF2-dependent activity in endothelial cells [20], [23].

PRL-3 expressing cancer cells are able to recruit endothelial cells for the initiation of tumor angiogenesis [24]. It has also been suggested that PRL-3 induces microvascular and lymphatic formation in lung cancer tissues associated with elevated VEGF expression [25]. *PRL-3* transcript is abundant both in malignant tumor and metastatic lesions, but also in vasculature and endothelial cells within a malignant tumor mass [2], [7], [10], [26], [27], [28], [29]. PRL-3 therefore appears to play a role in tumor-associated endothelial cells, but neither the control of *PRL-3* mRNA expression in tumor endothelial cells nor the significance of PRL-3 over-expression for the function of these cells is well understood. In normal mouse and human tissues, *PRL-3* transcript is found predominantly in skeletal muscle and heart [1], [24], [30], but the identity of the controlling transcription factors is not well defined. Although Basak et al. [31] reported that mouse *prl-3* gene was a direct target of P53 during DNA damage-induced cell cycle arrest, considering its expression pattern in normal tissues, it is quite possible that the transcription of human *PRL-3* is under the control of cardiac- or muscle-specific transcription factors.

Here, we demonstrate that VEGF can induce *PRL-3* transcription in HUVEC through the transcription factor MEF2C, and that both PRL-3 inhibitor and siRNA-induced knockdown can be used to suppress the tube formation by HUVEC. We suggest on this basis that PRL-3 is a downstream component of the VEGF/MEF2C pathway in endothelial cells, and that it may play an important role in tumor angiogenesis.

## Materials and Methods

### Cell culture

HEK293T, Hela, A375, A549, MCF7, SW480 cells, all obtained from the Institute of Biochemistry and Cell Biology (Shanghai, China), were cultured in DMEM supplemented with penicillin (100 U/ml), streptomycin (100 µg/ml) and 10% v/v FBS (Life Technologies Inc., Grand Island, NY). HUVEC (provided by Decai Yu in Gulou Hospital, Nanjing, China) were cultured in EGM2-MV medium (Lonza) and used at passages 4 to 12. Cells were grown in 5% v/v CO_2_ at 37°C, and the medium was replaced every 2–3 days.

### Localization of the *PRL-3* transcription start site

The *PRL-3* transcription start site was located in HUVEC, 293T and Jurkat cells using a GeneRacer kit (Invitrogen), according to the manufacturer's instructions. The initial 5′RACE PCR used cDNAs as templates, primed with the 5′ primer supplied with the kit and *PRL-3* primer GSP (5′-CCAAAGTAAAGCGGGCAACTCCAA-3′). The amplification regime comprised an initial denaturation step (94°C/5min), followed by 30 cycles of 94°C/30s, 60°C/30s, 72°C/45s, ending with an elongation step of 72°C/5min. The second PCR used the primary PCR products as templates, primed with the 5′ nested primer supplied with the kit and the *PRL-3* primer nested GSP (5′-GCAACTCCAAACTCCCGTCTCTC-3′). The amplification regime was identical to the above, except that the annealing temperature was set to 65°C rather than 60°C. The amplicon was gel-purified using a gel extraction kit (OMEGA, USA) and cloned into the TOPO-TA Cloning vector (Invitrogen) for subsequent sequencing.

### Construction of luciferase reporter plasmids

A series of *PRL-3* promoter plasmids were constructed by cloning the *PRL-3-iso2* promoter region into the pGL3 basic vector (Promega, Madison, WI, USA), and their names are based on the position of the inserted sequence's 5′ most nucleotide relative to the transcription start site. Briefly, a DNA fragment containing the *PRL-3* promoter region was amplified from HUVEC DNA and cloned into the pGL3 basic vector using primers in Table S1. Recombination PCR was performed as described previously [32] to introduce mutations into the MEF2 binding sites based on luc-158, using the primers listed in Table S2. Briefly, the products amplified by primer pairs P1 and P2 (or P3 and P4) were ligated to one another using a two-step PCR procedure. The ligated sequence was then inserted into the pGL3 vector.

### Plasmid constructs

The MEF2C coding region was amplified from total RNA isolated from human skeletal muscle using the following primer pair: 5′-GGAGCTAGCGGACTATGGGGAGAAAAA-3′ and 5′- ACAGAGCTCATCATGTTGCCCATCCTT-3′. The resulting amplicon was inserted into the PcDNA3.1 (−) *Nhe*I*/Xho*I cloning site. MEF2-DBD-VP16-ER and MEF2-ΔDBD-VP16-ER (the latter lacking the core residues responsible for MEF2 DNA binding) are kindly provided by Dr. Michael Greenberg [33]. The ER sequence ensures that MEF2-DBD-VP16-ER remains in the cytoplasm of the transfected cells and rapidly translocates into the nucleus to activate MEF2-dependent transcription upon 4-hydroxyl-tamofxifen (4OHT) stimulation. MEF2-DBD-VP16 and MEF2-ΔDBD-VP16 were subcloned from MEF2-DBD-VP16-ER and MEF2-ΔDBD-VP16-ER into the PcDNA3.1 (−) vector, while MEF2-DBD-NLS and MEF2-ΔDBD-NLS represented fusions of the nuclear localization signal (NLS) domain of MEF2C with either the MEF2-DBD or MEF2-ΔDBD domain of, respectively, MEF2-DBD-VP16-ER and MEF2-ΔDBD-VP16-ER.

### Transfection and luciferase assay

HEK293T cells were cultured in 24-well plates and transiently transfected with the *PRL-3* promoter reporters using Lipofectamine 2000 (Invitrogen). The transfection process involved the introduction of 0.5 µg luciferase reporter plasmid and 0.1 µg of β-gal vector; MEF2 plasmids were included where necessary. After 48 h, luciferase activity was detected using the Luciferase Assay System (Promega) and β-Galactosidase using the β-Galactosidase Enzyme Assay System (Promega), both according to the manufacturer's instructions. Each transfection was carried out in triplicate. For the knockdown assay, either 200 pM of a non-targeting siRNA or MEF2C-specific siRNA (Stealth™ RNA, Invitrogen), or various concentrations of *PRL-3*-specific siRNA, was transfected into cells using Lipofectamine 2000. The sequences of *MEF2C* siRNAs (#1, #2, #3) and *PRL-3* siRNA are listed in Table S5.

### Electrophoretic mobility shift assay (EMSA)

Nuclear extracts were prepared from HEK293T cells transfected with the MEF2C plasmid using NE-PER nuclear and cytoplasmic extraction reagents (Pierce, Rockford, IL, USA) according to the manufacturer's instructions. Synthetic oligonucleotides 5′-labeled with biotin (Invitrogen) were annealed to generate double-stranded oligonucleotides as probes (Table S3). EMSA was performed according to the instructions provided with the LightShift Chemiluminescent EMSA kit (Pierce). Briefly, 1 µl of nuclear extract was incubated at room temperature for 20 min in the presence of 1 µl binding buffer, 20 fmol of biotinylated probe, 0.5 µl glycine, 0.5 µl MgCl2, 0.5 µl NP40 and 1 µl Poly(dI.dC) in a reaction volume of 10 µl. Then 2.5 µl of 5×loading buffer was added and the sample electrophoresed through a 4% non-denaturing polyacrylamide gel in 0.5×TBE at 4°C. For competition experiments, unlabelled wild-type oligonucleotide was added in a 100× molar excess prior to the addition of the biotinylated probe. To identify the transcription factor present in the DNA protein complex using a supershift assay, the nuclear extracts were incubated in the binding buffer for 60 min at 4°C in the presence of MEF2C or IgG antibody (Santa Cruz Biotechnology, Santa Cruz, CA, USA) prior to the addition of the biotinylated probe.

### Chromatin Immunoprecipation assay

The ChIP assay was performed as described previously [34]. Either anti-MEF2C or anti-IgG antibodies were used for immunoprecipation. The primers used to amplify the region containing MEF2 binding sites were 5′-CGGCGGGAGGAAGGAGGGGT-3′ and 5′-GCTGCCGCCACCGCCGCCTG-3′.

### RT-PCR and Real-time PCR

A 1 µg aliquot of total RNA extracted from cells with the Trizol reagent (Invitrogen, Carlsbad, CA) was converted to cDNA using M-MLV reverse transcriptase (Toyobo, Osaka, Japan) according to the manufacturer's instructions. RT-PCR was performed using primers listed in Table S4. Real-time PCR was performed on an ABI Prism 7300 system device (Applied Biosystems, Foster City, CA), using SYBR Green I dye (Biotium, Inc.).

### Western blot analysis

The Western blotting procedure followed standard protocols. HEK293T cells, after being transfected with plasmids or siRNA as indicated for 48 hours, were collected and lysed in 50 mM Tris (pH 8.0), 150 mM NaCl, 1% NP-40, 0.1% SDS, 5 mM EDTA, 0.1 mM PMSF, 0.15 U/mL aprotinin and 1 µg/mL pepstatin. Anti-PRL-3 (kindly provided by Zeng Qi, Institute of Molecular and Cell Biology, Singapore, Singapore), anti-MEF2C and anti-GAPDH (Santa Cruz Biotechnology) antibodies were used for detection.

### HUVEC tube formation assay

A tube formation assay was performed as described previously [35]. Briefly, 2×10^4^ HUVEC were seeded in triplicate in a 96-well plate pre-coated with Matrigel (BD) in EGM2 medium in either the presence or absence of various concentrations of PRL-3 inhibitor P0108 (Sigma) at 37°C in a 5% v/v CO_2_ humidified atmosphere. For the PRL-3 knockdown assay, cells were transfected with various concentrations of *PRL-3* specific siRNA for 48 h, and then seeded in triplicate in a 96-well plate. After 24 h, cell images were captured and analyzed.

### Statistical analysis

Data were expressed as mean ± SD. The student's *t*-test was used to attach statistical significance to differences between means, applying a P<0.05 or 0.01 criterion.

## Results

### VEGF activates *PRL-3* transcription in HUVEC

**Figure 1:**
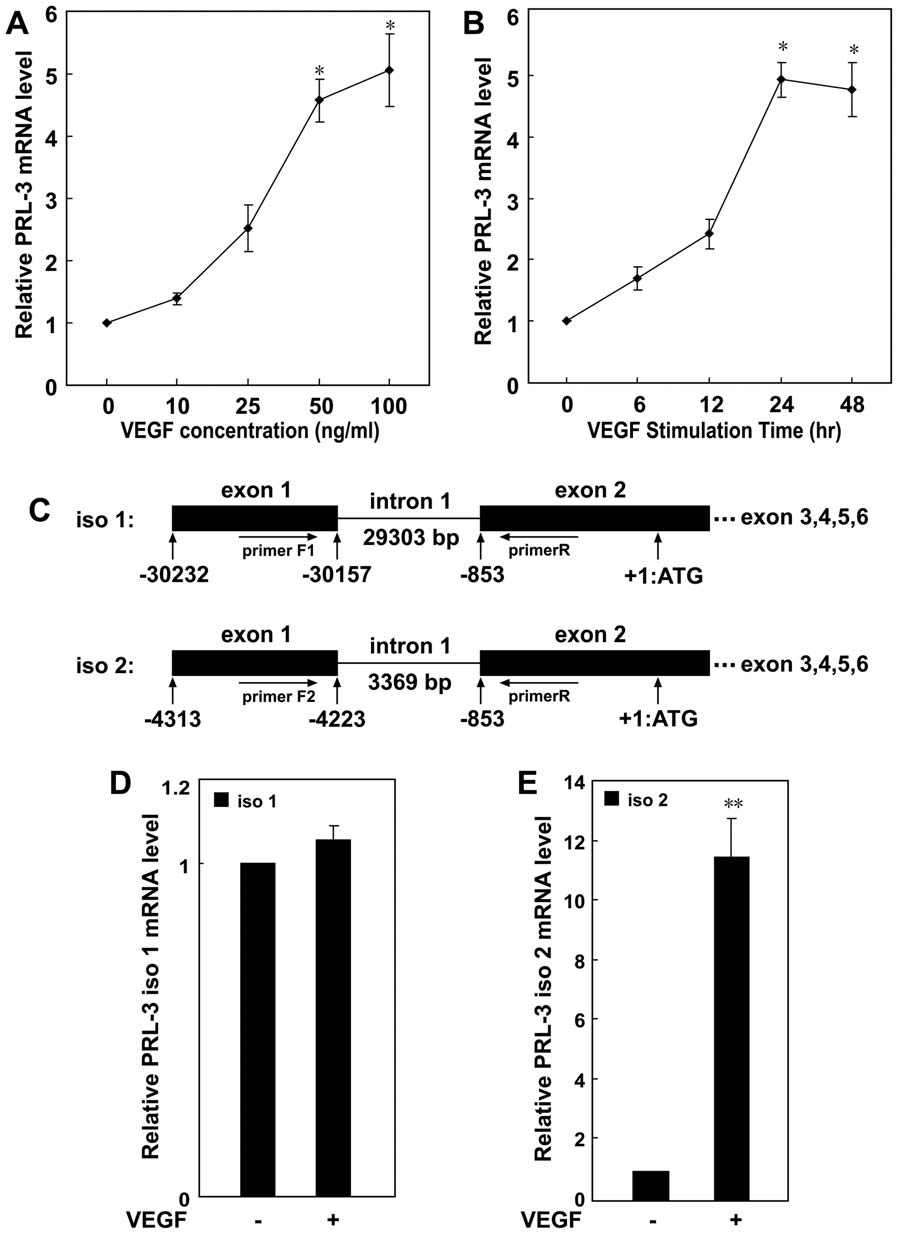
VEGF activates *PRL-3* transcription in HUVEC. (**A**) HUVEC were stimulated by the addition of various concentrations of VEGF for 24 h. (B) The time course of *PRL-3* transcription in response to 100 ng/ml VEGF. *RPL-3* mRNA level was evaluated by real-time PCR. Control is the untreated cells. (**C**) Schematic of the *PRL-3* gene indicating the transcription initiation sites and its exon/intron structure. VEGF selectively increases the abundance of the *PRL-3-iso2* (**E**) but not the *PRL-3-iso1* (**D**) transcript in HUVEC treated with 100 ng/ml VEGF for 24 h. The expression (real-time PCR) data reflect the mean ± SD from three independent experiments. * significantly different from the control at P<0.05.

Because *PRL-3* is highly expressed in the vasculature and endothelial cells of malignant tumor mass and its protein can only be detected in developing blood vessels, it is interesting to explore whether VEGF, a growth factor proven to be important for blood vessel formation, could induce *PRL-3* expression in endothelial cells. When HUVEC were exposed to either 50 ng/ml or 100 ng/ml VEGF for 24 h, the abundance of *PRL-3* transcript rose significantly (Fig. 1A). The level plateaued after 24 h, remaining high for the following 24 h (Fig. 1B). The *PRL-3* transcription start site was obtained using the 5′RACE method. After cloning its 5′UTR (sequence given in Supplementary Sequence S1) the alignment of the resulting sequence suggested two distinct transcription start sites, one lying 30232 bp and the other 4313 bp upstream of the ATG codon. As a result, two distinct *PRL-3* transcripts were predicted (*PRL-3-iso1* and *PRL-3-iso2*) which differ with respect to their first exon and intron (Fig. 1C). Using primers specific to each transcript, it could be shown that VEGF selectively induced the transcription of *PRL-3-iso2*, while it had no effect on the level of *PRL-3-iso1* transcription (Fig. 1D and E).

### MEF2 binding sites are critical for the promoter activity of *PRL-3*

**Figure 2:**
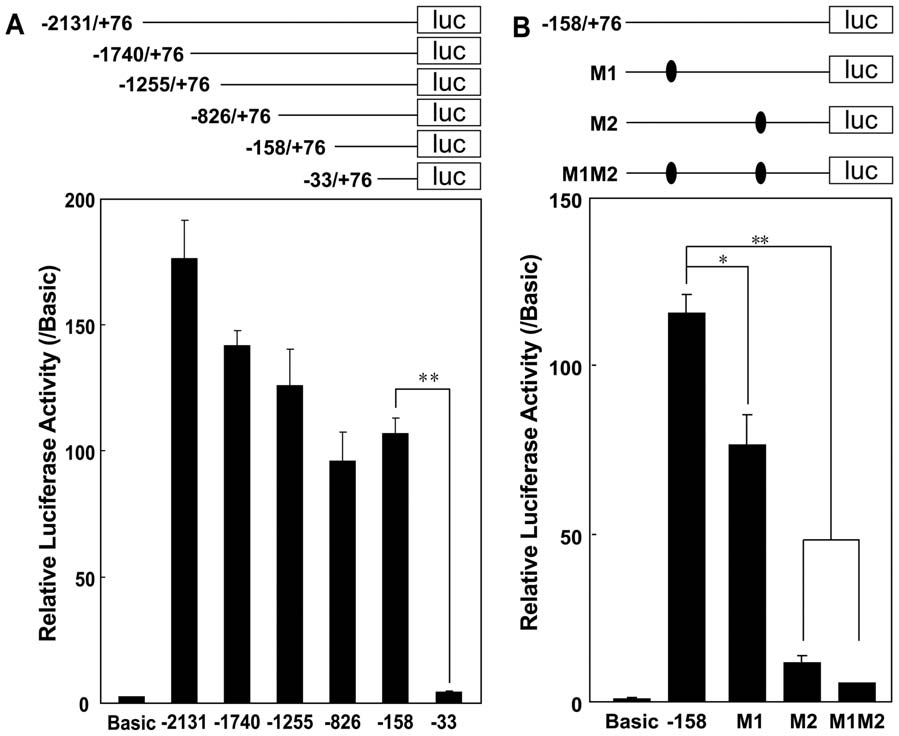
MEF2 binding sites are critical for the promoter activity of *PRL-3*. (**A**) The region between −158 and −33 is important for the transcriptional activity of the *PRL-3* promoter, as measured by luciferase activity. (**B**) The effect of site-directed mutations in the MEF2 binding sites, as measured by luciferase activity. All luciferase activity were normalized to β-galactosidase activity and standardized to the normalized activity of pGL3-Basic. Each column represents the mean ± SD of three independent experiments. ** significantly different from the control at P<0.01.

Next, the *PRL-3-Iso2* promoter region between 2131 bp upstream and either 76 bp or 603 bp downstream of the transcription start site was cloned into a luciferase reporter construct. The two constructs induced a similar high level of luciferase activity in 293T cells (Fig. S1), indicating that the region upstream of +76 determined all the transcriptional activity. A series of truncated *PRL-3* promoter region constructs were therefore assembled; the level of luciferase activity decreased to almost zero as the 5′ truncation point was moved from to −2131 to −1740, −1255, −826 and −158 and finally to −33 (Fig. 2A). The sequence between −158 and −33 was queried using www.genomatix.de, revealing the presence of two MEF2 binding sites: M1 lying between −128 and −119 (CTATATTTAG) and M2 from −49 to −40 (CTATAAATAG). As *PRL-3* transcript is abundant in heart and skeletal muscle where the presence of MEF2 is known to be critical, it appears likely that the deletion of the two MEF2 binding sites was responsible for the above observed transcriptional activity. To verify this hypothesis, site-directed mutations were introduced into the M1 and M2 sequences, and these did indeed result in a drastic decrease in luciferase activity (Fig. 2B). Thus we concluded that the MEF2 binding sites are important for the transcriptional activity of the *PRL-3* promoter.

### MEF2C binds to *PRL-3* promoter *in vitro* and *in vivo*

**Figure 3:**
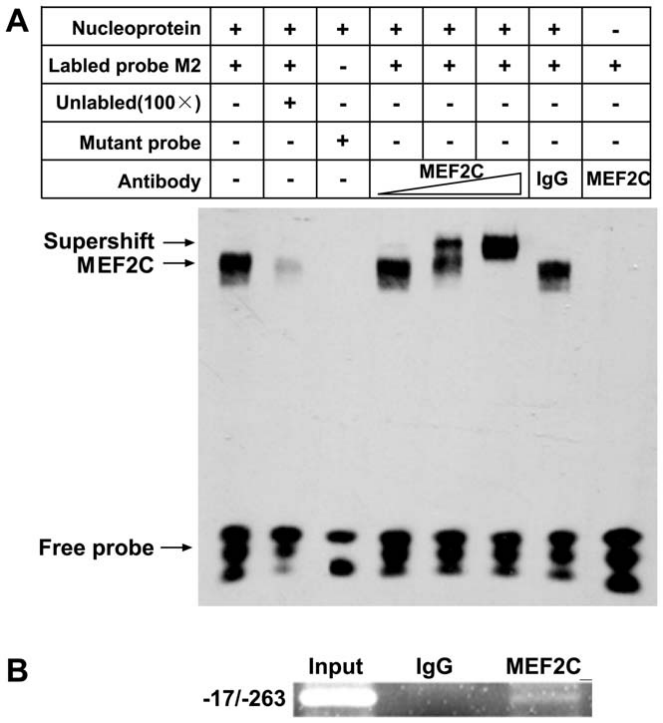
MEF2C binds to the *PRL-3* promoter both *in vitro* and *in vivo*. (**A**) MEF2C binds *in vitro* to the MEF2 binding site M2 in the promoter region of *PRL-3-iso2*. The EMSA used MEF2-M2 wild-type and mutated probes and nuclear extracts from 293T cells transfected with PcDNA3.1-MEF2C. (**B**) MEF2C binds to the *PRL-3* promoter region *in vivo*. The crosslinked DNA-protein complex extracted from 293T cells was sonicated and immunopreciptated with either anti-IgG or anti-MEF2C antibody, and then the DNA present in the immunoprecipitate was extracted and used as a template for PCR. 1 µl of sonicated chromatin was used as template for the input. The data are representative of three independent experiments.

EMSA was applied to establish whether the two MEF2 binding sites were able to bind MEF2C. DNA-protein complexes were detected when wild-type labeled MEF2-M2 probes were used (Fig. 3A, lane 1) but not when using mutated probes (lane 3). The complexes formed were competitively blocked in the presence of excess unlabeled probes (lane 2). Adding a variable quantity (0.008 µg, 0.04 µg, 0.2 µg) of anti-MEF2C antibody induced the formation of a supershift band in a dose-dependent manner (lane 4–6), which was not inducible by the addition of an IgG antibody (lane 7). The supershift band was not generated by the addition of just the MEF2C antibody (lane 8). MEF2C also bound successfully to both MEF2-M1 and MEF2-M2 sites, although the binding ability to the former site was less strong (Fig. S2). The same interaction was investigated *in vivo* using the ChIP assay. As shown in figure 3B, the anti-MEF2C antibody specifically immunoprecipitated the *PRL-3* promoter in 293T cells while the IgG antibody did not (Fig. 3B).

### MEF2C promotes the transcriptional activity of the *PRL-3* promoter

**Figure 4:**
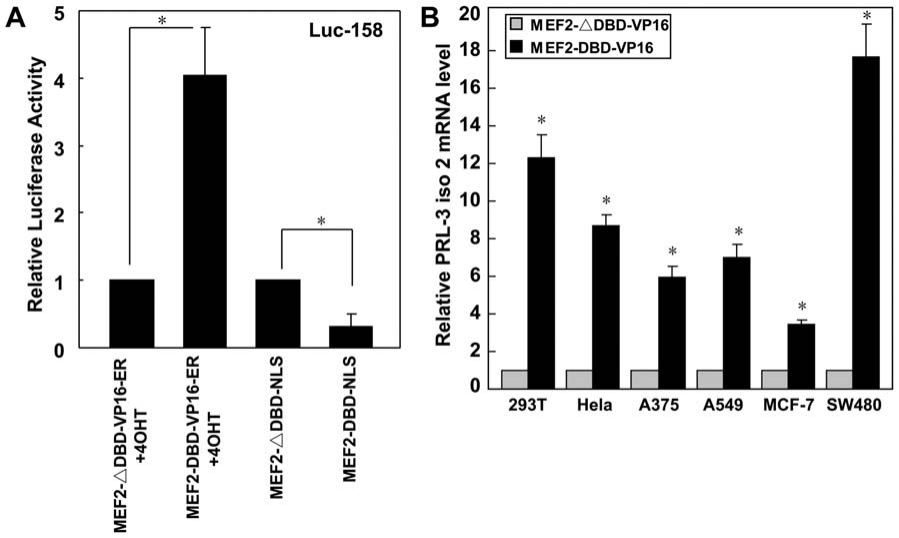
Human *PRL-3* transcription is regulated by MEF2C. (**A**) The effect of MEF2 plasmid on the luciferase activity of luc-158. Twenty-four hours after transfection, 293T cells were stimulated with 4-OHT for another 24 h and harvested for luciferase assay. (**B**) The constitutively active form of MEF2C up-regulated *PRL-3-iso2* mRNA in several human cell lines. Cells were transfected with the indicated plasmids for 48 h and *PRL-3* mRNA level was evaluated by real-time PCR.

To determine whether *PRL-3* expression can be regulated by MEF2C, the constitutively active form of MEF2C (MEF2-DBD-VP16-ER) and the dominant negative MEF2C (MEF2-DBD-NLS), were introduced in conjunction with a luciferase assay. The luciferase activity was greatly increased by the former in the presence of 4OHT, while it was markedly reduced by the latter (Fig. 4A). In several human cell lines, the former also increased the abundance of the *PRL-3-Iso2* transcript, but had no effect on the abundance of the *PRL-3-Iso1* transcript (Fig. 4B, S3). However, there was no concomitant increase in the abundance of the protein product (Fig. S4), indicating that the translation of *PRL-3* is strictly regulated.

### MEF2C is required for VEGF- induced transcription of *PRL-3* in HUVEC

**Figure 5:**
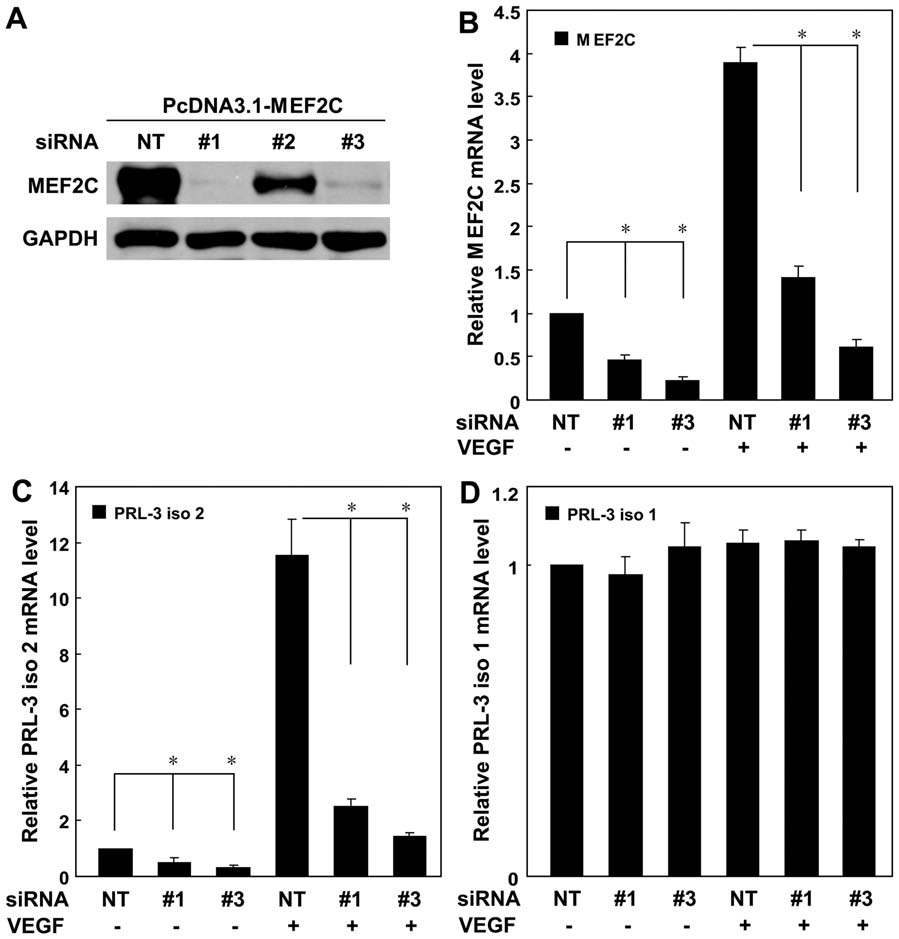
MEF2C is required for VEGF-induced production of the *PRL-3-iso2* transcript in HUVEC. (**A**) The 293T cell line was co-transfected with PcDNA3.1-MEF2C and siRNAs targeting either MEF2C or a control (NT) sequence. Twenty-four hours later, cells were collected and lysed, and then protein levels of MEF2C and GAPDH were detected by Western blot analysis. (**B**, **C**, **D**) Knockdown of MEF2C suppresses the production of endogenous and VEGF-induced MEF2C and the *PRL-3-iso2* transcript, but has no effect on the abundance of the *PRL-3-iso1* transcript. HUVEC were transfected with #1 and #3 siRNAs specific for MEF2C or a non-targeting sequence (NT). Forty-eight hours after transfection, cells were treated with 100 ng/ml VEGF for another 24 h and then assessed for gene expression using real-time-PCR. The data reflect the mean ± SD from three independent experiments. * significantly different from the control at P<0.05.

Since VEGF induces the expression of MEF2C, it was of interest to explore the role of VEGF in determining *PRL-3* transcription mediated by MEF2C. For this purpose, a siRNA strategy was employed to knockdown the production of MEF2C in HUVEC, and then to stimulate these cells with VEGF. Two siRNA oligoes (#1 and #3) successfully reduced the transcription of both constitutive and VEGF-induced MEF2C (Fig. 5B) and *PRL-3-Iso2* (Fig. 5C), but had no effect on that of *PRL-3-Iso1* (Fig. 5D). We concluded that VEGF can indeed induce the production of the *PRL-3-Iso2* transcript in HUVEC through transcription factor MEF2C.

### *PRL-3* is important for tube formation of HUVEC

**Figure 6:**
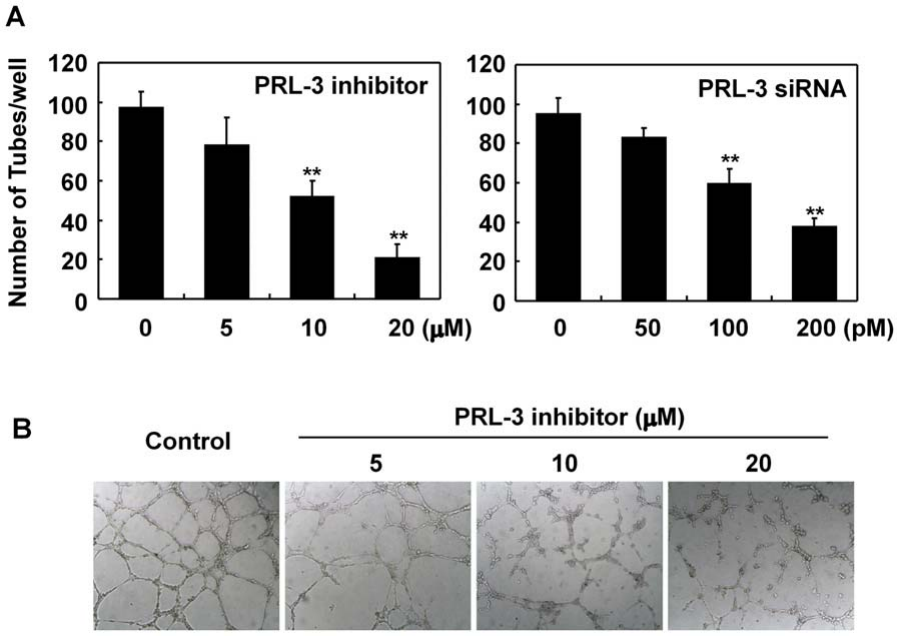
The presence of PRL-3 is required for tube formation by HUVEC cultured on matrigel. (**A**) HUVEC were seeded into 6-well plates precoated with matrigel in EGM2 medium in the presence of various concentrations of PRL-3 inhibitor (left panel). HUVEC were transfected with various concentrations of human *PRL-3* specific siRNA for 48 h, and then cells were harvested and seeded into 6-well plates precoated with matrigel in EGM2 medium (right panel). (**B**) Representative images from three independent experiments.

Finally, we tried to investigate the role of PRL-3 in an endothelial cell function, tube formation. When the function of PRL-3 was disrupted either by the presence of the specific inhibitor P0108, or by the siRNA-induced knockdown of *PRL-3* expression, tube formation by HUVEC was compromised (Fig. 6). This behavior was consistent with the observation that over-expression of PRL-3 in HUVEC promotes tube formation [28].

## Discussion

PRL-3 is a metastasis-associated phosphatase whose ectopic expression can markedly enhance cancer cell invasion and migration. The gene is highly expressed in tumor associated endothelial cells, including vasculature and endothelial cells in colorectal cancer [26], glioma tumor tissue [29] and invasive breast cancer [27]. It is implicated in the enhancement of the migration capacity of endothelial cells, at least *in vitro* [7]. Here, we have shown that VEGF can induce *PRL-3* transcription in HUVEC, mediated by the transcription factor MEF2C, which suggests the existence of a novel VEGF/MEF2C/PRL-3 signaling pathway in endothelial cells.

*PRL-3* possesses two distinct transcription start sites (Fig. 1C), which suggests that its transcription is controlled by two independent promoters. The *PRL-3* promoter region suggested by the GeneBank entry (NM_032611) [36], [37], in reality lies within the gene's first intron. As VEGF selectively induced the production of the *PRL-3-iso2* transcript, only the second promoter was sensitive to VEGF stimulation (Fig. 1D, E). Possibly therefore, the *PRL-3-iso1* transcript represents the constitutive level of *PRL-3* expression while the *PRL-3-iso2* transcript is VEGF-inducible. This could explain the high abundance of *PRL-3* transcript in tumor-associated endothelial cells, since tumor cells are able to release VEGF into the extracellular matrix [16]. Experimentally, it was possible to detect a substantial level of VEGF in the supernatant after a 24 h culturing of the colon cancer cell lines SW480 and SW620 (data not shown).

In normal mouse and human tissues, *PRL-3* mRNA is found predominantly in skeletal muscle and heart [1], [24], [30], which implies that the gene is regulated by cardiac- or muscle-specific transcription factors. Here, two functional MEF2 binding sites have been located in its promoter region (Fig. 2B, Fig. 3). Coincidently perhaps, the promoter region of *Nur77*, a gene known also to be regulated by the MEF2 family [38], [39], also possesses two MEF2 consensus binding sites [40]. Thus it is conceivable that MEF2 plays a key role in *PRL-3* transcription. The constitutively active form of MEF2C increased *PRL-3* transcription, and enhanced the abundance of both *PRL-3-iso2* and *Nur77* transcripts in several human cell lines (Fig. 4 and data not shown). Although *PRL-3* transcription was clearly enhanced by the presence of MEF2C, there was no equivalent enhancement of the protein level (Fig. S3). It has been reported recently that the abundance of PRL-3 protein is not directly associated with the level of its transcript, and that PCBP1 can suppress *PRL-3* translation by binding to its 5′UTR [36]. In addition, FKBP38 is known to promote the degradation of endogenous PRL-3 protein [41]. We assume that both the suppression of translation and the promotion of degradation may be released in the micro-environment conditions existing within a malignant tumor or metastases site, thereby allowing the VEGF-induced *PRL-3* transcripts to be readily translated.

Using a knockdown strategy, it was possible to show that VEGF-induced *PRL-3* transcription is mediated by MEF2C (Fig. 5), implying the existence in endothelial cells of an as yet undescribed VEGF/MEF2C/PRL-3 signaling pathway. PRL-3 is known to induce microvascular and lymphatic formation in lung cancer tissues, and this phenomenon has been associated with an increased level of VEGF expression [25]. Either the addition of a PRL-3 inhibitor, or the reduction in PRL-3 levels obtained by siRNA knockdown, inhibited tube formation in *in vitro* cultured HUVEC (Fig. 6). Together with the finding that PRL-3 contributes to angiogenesis in tumors [25], we suggest that cancer cells containing an abundance of PRL-3 tend to more freely release VEGF into the extracelluar matrix, which acts to stimulate PRL-3 expression in endothelial cells via the VEGF/MEF2C pathway, and helps to initiate tumor angiogenesis. Thus, PRL-3 may represent a rational drug target for cancer therapy, because in attempting to neutralize PRL-3, not just the cancer cells themselves, but also their associated endothelial cells are targeted.

**Figure 7:**
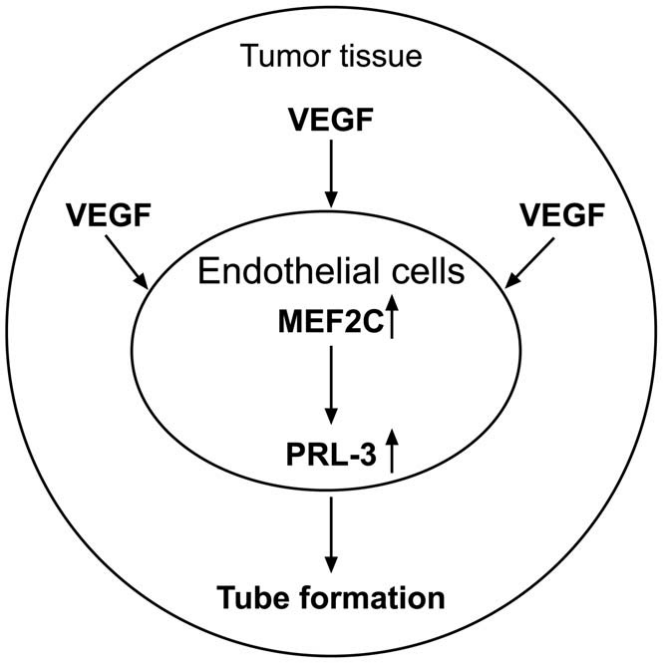
Hypothesized schematic diagram for VEGF/MEF2C/PRL-3 signaling in endothelial cells. In malignant tumor tissues, endothelial cells can be exposed to VEGF secreted from the cancer cells. VEGF induces the expression and activity of MEF2C, which elevates *PRL-3* transcription in the endothelial cells. Then PRL-3 promotes tube formation or angiogenesis in the endothelial cells within the tumor tissue.

In conclusion, we have shown that the transcription of human *PRL-3* is initiated from two distinct sites, resulting in the formation of two novel *PRL-3* transcripts. VEGF selectively induces the production of the *PRL-3-iso2* transcript mediated by the transcription factor MEF2C, suggesting an important role for both MEF2C and PRL-3 in endothelial cells function, and in particular in tumor angiogenesis (Fig. 7). We hope that these findings will facilitate further investigation of the mechanisms underlying the regulation of *PRL-3* expression, and provide a better understanding of the role of PRL-3.

## Supporting Information

Figure S1 Regions upstream of the transcription start site determine most of the transcriptional activity of the *PRL-3* promoter. The promoter constructs (containing regions from −2131 to either +603 or +76 relative to the transcription start site) were co-transfected with a β-gal plasmid into HEK293T cells. Luciferase activity in the transfected cells was measured after 48 h. Each column represents the mean ± SD of three independent experiments. ** significantly different from the control at P<0.01.
(TIF)

Figure S2 MEF2C binds to the MEF2 binding site M1 in the promoter of *PRL-3-iso2*. EMSA was performed using MEF2-M1 wild-type and mutated probes and nuclear extracts from 293T cells transfected with PcDNA3.1-MEF2C plasmid. Data shown are representative of three independent experiments.
(TIF)

Figure S3 The constitutively active form of MEF2C has no effort on the abundance of the *PRL-3-iso1* transcript. Cells were transfected with the plasmids indicated for 48 h and the abundance of the *PRL-3-iso1* transcript was evaluated by real-time PCR. The expression data reflect mean ± SD from three independent experiments.
(TIF)

Figure S4 The constitutively active form of MEF2 has no effect on the abundance of the PRL-3 protein. The 293T cell line was transfected with the plasmids indicated for 48 h, and the abundance of PRL-3 and GAPDH was estimated by Western blots. Data shown are representative of three independent experiments.
(TIF)

Supplementary Sequence S1 (DOC)

Table S1 Primers used for amplification of constructs of the *PRL-3 promoter.*
(DOC)

Table S2 Primers used for Recombination PCR.
(DOC)

Table S3 The sequences of MEF2 probes used for EMSA.
(DOC)

Table S4 Primers used for RT-PCR and Real-time PCR.
(DOC)

Table S5 siRNA sequences used for MEF2C and PRL-3 knockdown experiments.
(DOC)

## References

[1] Zeng Q, Hong W, Tan YH (1998). Mouse PRL-2 and PRL-3, two potentially prenylated protein tyrosine phosphatases homologous to PRL-1.. Biochem Biophys Res Commun.

[2] Saha S, Bardelli A, Buckhaults P, Velculescu VE, Rago C (2001). A phosphatase associated with metastasis of colorectal cancer.. Science.

[3] Stephens BJ, Han H, Gokhale V, Von Hoff DD (2005). PRL phosphatases as potential molecular targets in cancer.. Mol Cancer Ther.

[4] Miskad UA, Semba S, Kato H, Yokozaki H (2004). Expression of PRL-3 phosphatase in human gastric carcinomas: close correlation with invasion and metastasis.. Pathobiology.

[5] Wu X, Zeng H, Zhang X, Zhao Y, Sha H (2004). Phosphatase of regenerating liver-3 promotes motility and metastasis of mouse melanoma cells.. Am J Pathol.

[6] Polato F, Codegoni A, Fruscio R, Perego P, Mangioni C (2005). PRL-3 phosphatase is implicated in ovarian cancer growth.. Clin Cancer Res.

[7] Radke I, Gotte M, Kersting C, Mattsson B, Kiesel L (2006). Expression and prognostic impact of the protein tyrosine phosphatases PRL-1, PRL-2, and PRL-3 in breast cancer.. Br J Cancer.

[8] Wang Y, Li ZF, He J, Li YL, Zhu GB (2007). Expression of the human phosphatases of regenerating liver (PRLs) in colonic adenocarcinoma and its correlation with lymph node metastasis.. Int J Colorectal Dis.

[9] Liu YQ, Li HX, Lou X, Lei JY (2008). Expression of phosphatase of regenerating liver 1 and 3 mRNA in esophageal squamous cell carcinoma.. Arch Pathol Lab Med.

[10] Sager JA, Benvenuti S, Bardelli A (2004). PRL-3: a phosphatase for metastasis?. Cancer Biol Ther.

[11] Bessette DC, Qiu D, Pallen CJ (2008). PRL PTPs: mediators and markers of cancer progression.. Cancer Metastasis Rev.

[12] Al-Aidaroos AQ, Zeng Q (2010). PRL-3 phosphatase and cancer metastasis.. J Cell Biochem.

[13] Zeng Q, Dong JM, Guo K, Li J, Tan HX (2003). PRL-3 and PRL-1 promote cell migration, invasion, and metastasis.. Cancer Res.

[14] Kato H, Semba S, Miskad UA, Seo Y, Kasuga M (2004). High expression of PRL-3 promotes cancer cell motility and liver metastasis in human colorectal cancer: a predictive molecular marker of metachronous liver and lung metastases.. Clin Cancer Res.

[15] Fagerli UM, Holt RU, Holien T, Vaatsveen TK, Zhan F (2008). Overexpression and involvement in migration by the metastasis-associated phosphatase PRL-3 in human myeloma cells.. Blood.

[16] Yancopoulos GD, Davis S, Gale NW, Rudge JS, Wiegand SJ (2000). Vascular-specific growth factors and blood vessel formation.. Nature.

[17] Ambler CA, Schmunk GM, Bautch VL (2003). Stem cell-derived endothelial cells/progenitors migrate and pattern in the embryo using the VEGF signaling pathway.. Dev Biol.

[18] Han J, Molkentin JD (2000). Regulation of MEF2 by p38 MAPK and its implication in cardiomyocyte biology.. Trends Cardiovasc Med.

[19] McKinsey TA, Zhang CL, Olson EN (2002). MEF2: a calcium-dependent regulator of cell division, differentiation and death.. Trends Biochem Sci.

[20] Lin Q, Lu J, Yanagisawa H, Webb R, Lyons GE (1998). Requirement of the MADS-box transcription factor MEF2C for vascular development.. Development.

[21] Lin Q, Schwarz J, Bucana C, Olson EN (1997). Control of mouse cardiac morphogenesis and myogenesis by transcription factor MEF2C.. Science.

[22] Bi W, Drake CJ, Schwarz JJ (1999). The transcription factor MEF2C-null mouse exhibits complex vascular malformations and reduced cardiac expression of angiopoietin 1 and VEGF.. Dev Biol.

[23] Maiti D, Xu Z, Duh EJ (2008). Vascular endothelial growth factor induces MEF2C and MEF2-dependent activity in endothelial cells.. Invest Ophthalmol Vis Sci.

[24] Guo K, Li J, Wang H, Osato M, Tang JP (2006). PRL-3 initiates tumor angiogenesis by recruiting endothelial cells in vitro and in vivo.. Cancer Res.

[25] Ming J, Liu N, Gu Y, Qiu X, Wang EH (2009). PRL-3 facilitates angiogenesis and metastasis by increasing ERK phosphorylation and up-regulating the levels and activities of Rho-A/C in lung cancer.. Pathology.

[26] Bardelli A, Saha S, Sager JA, Romans KE, Xin B (2003). PRL-3 expression in metastatic cancers.. Clin Cancer Res.

[27] Parker BS, Argani P, Cook BP, Liangfeng H, Chartrand SD (2004). Alterations in vascular gene expression in invasive breast carcinoma.. Cancer Res.

[28] Rouleau C, Roy A, St Martin T, Dufault MR, Boutin P (2006). Protein tyrosine phosphatase PRL-3 in malignant cells and endothelial cells: expression and function.. Mol Cancer Ther.

[29] Kong L, Li Q, Wang L, Liu Z, Sun T (2007). The value and correlation between PRL-3 expression and matrix metalloproteinase activity and expression in human gliomas.. Neuropathology.

[30] Matter WF, Estridge T, Zhang C, Belagaje R, Stancato L (2001). Role of PRL-3, a human muscle-specific tyrosine phosphatase, in angiotensin-II signaling.. Biochem Biophys Res Commun.

[31] Basak S, Jacobs SB, Krieg AJ, Pathak N, Zeng Q (2008). The metastasis-associated gene Prl-3 is a p53 target involved in cell-cycle regulation.. Mol Cell.

[32] Horton RM, Cai ZL, Ho SN, Pease LR (1990). Gene splicing by overlap extension: tailor-made genes using the polymerase chain reaction.. Biotechniques.

[33] Flavell SW, Cowan CW, Kim TK, Greer PL, Lin Y (2006). Activity-dependent regulation of MEF2 transcription factors suppresses excitatory synapse number.. Science.

[34] Krieg AJ, Krieg SA, Ahn BS, Shapiro DJ (2004). Interplay between estrogen response element sequence and ligands controls in vivo binding of estrogen receptor to regulated genes.. J Biol Chem.

[35] Singh RP, Dhanalakshmi S, Agarwal C, Agarwal R (2005). Silibinin strongly inhibits growth and survival of human endothelial cells via cell cycle arrest and downregulation of survivin, Akt and NF-kappaB: implications for angioprevention and antiangiogenic therapy.. Oncogene.

[36] Wang H, Vardy LA, Tan CP, Loo JM, Guo K (2010). PCBP1 suppresses the translation of metastasis-associated PRL-3 phosphatase.. Cancer Cell.

[37] Jiang Y, Liu XQ, Rajput A, Geng L, Ongchin M (2011). Phosphatase PRL-3 is a direct regulatory target of TGFbeta in colon cancer metastasis.. Cancer Res.

[38] Black BL, Olson EN (1998). Transcriptional control of muscle development by myocyte enhancer factor-2 (MEF2) proteins.. Annu Rev Cell Dev Biol.

[39] Youn HD, Sun L, Prywes R, Liu JO (1999). Apoptosis of T cells mediated by Ca2+-induced release of the transcription factor MEF2.. Science.

[40] Shalizi A, Gaudilliere B, Yuan Z, Stegmuller J, Shirogane T (2006). A calcium-regulated MEF2 sumoylation switch controls postsynaptic differentiation.. Science.

[41] Choi MS, Min SH, Jung H, Lee JD, Lee TH (2011). The essential role of FKBP38 in regulating phosphatase of regenerating liver 3 (PRL-3) protein stability.. Biochem Biophys Res Commun.

