# Supplementary figures and images for: VEGF Promotes the Transcription of the Human PRL-3 Gene in HUVEC through Transcription Factor MEF2C
Source: PLoS One. 2011 Nov 2;6(11):e27165. doi: 10.1371/journal.pone.0027165 (PMC3206935; doi:10.1371/journal.pone.0027165)

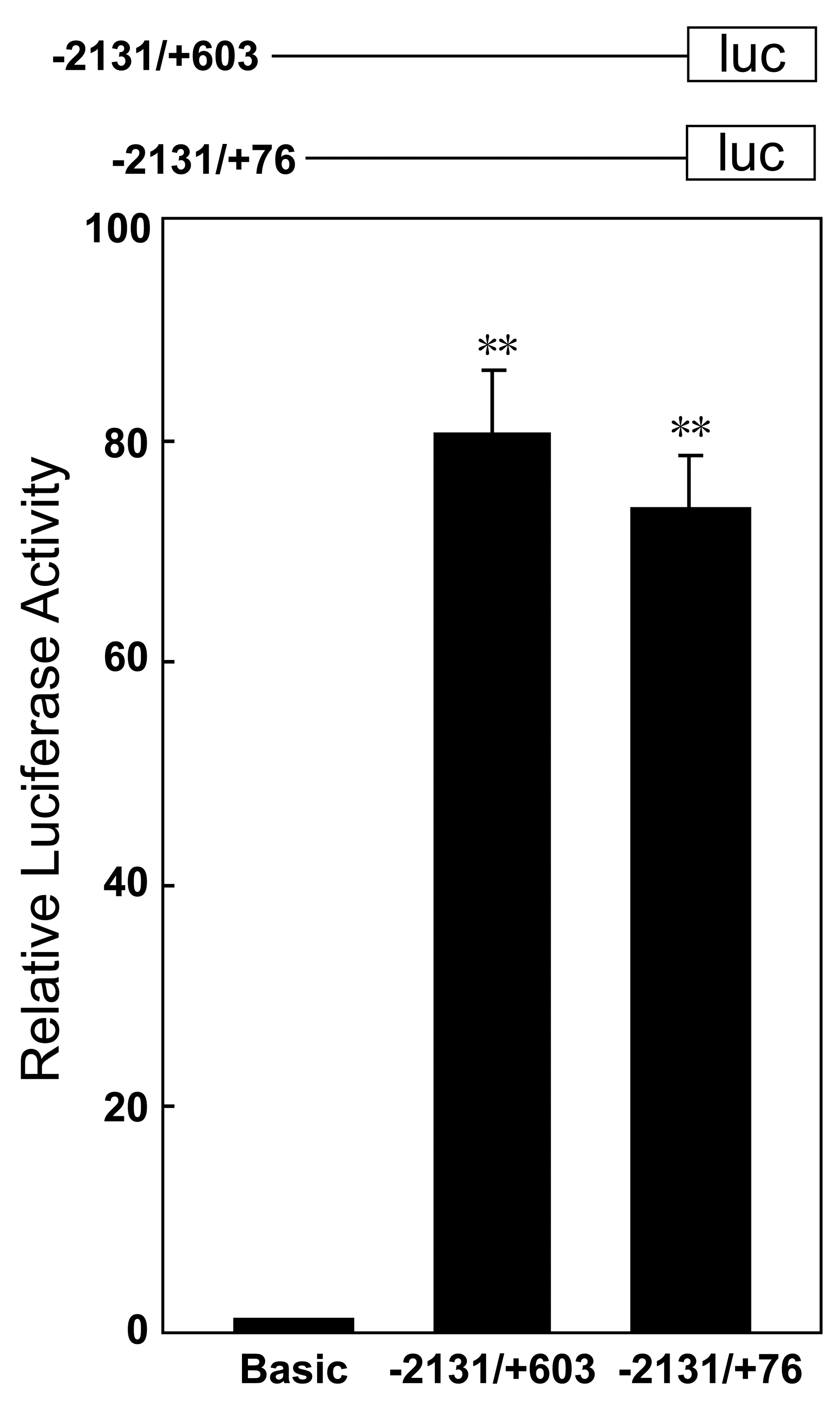

Supplement: Figure S1 — Regions upstream of the transcription start site determine most of the transcriptional activity of the PRL-3 promoter. The promoter constructs (containing regions from −2131 to either +603 or +76 relative to the transcription start site) were co-transfected with a β-gal plasmid into HEK293T cells. Luciferase activity in the transfected cells was measured after 48 h. Each column represents the mean ± SD of three independent experiments. ** significantly different from the control at P<0.01. (TIF) [file pone.0027165.s001.tif]

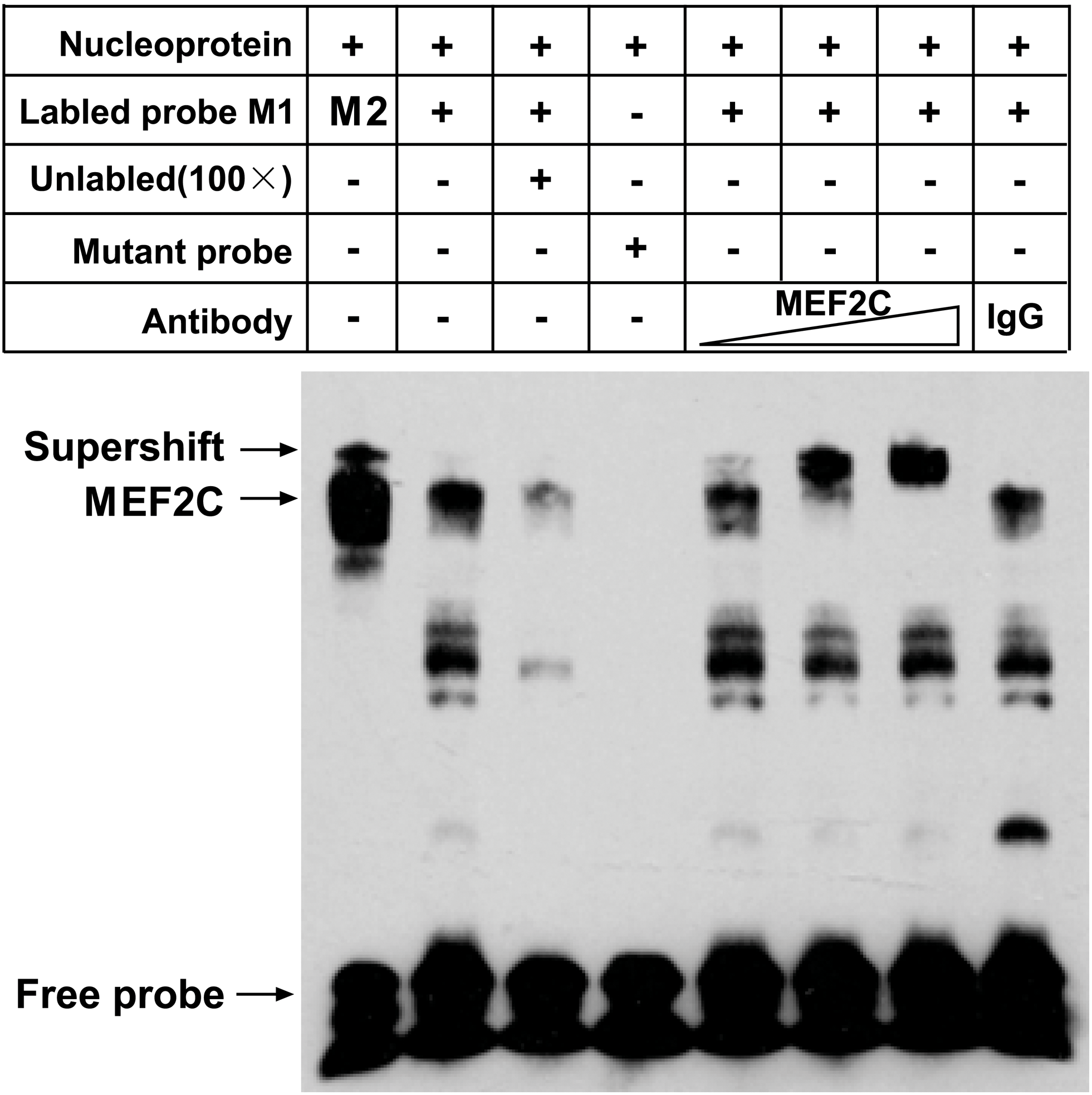

Supplement: Figure S2 — MEF2C binds to the MEF2 binding site M1 in the promoter of PRL-3-iso2. EMSA was performed using MEF2-M1 wild-type and mutated probes and nuclear extracts from 293T cells transfected with PcDNA3.1-MEF2C plasmid. Data shown are representative of three independent experiments. (TIF) [file pone.0027165.s002.tif]

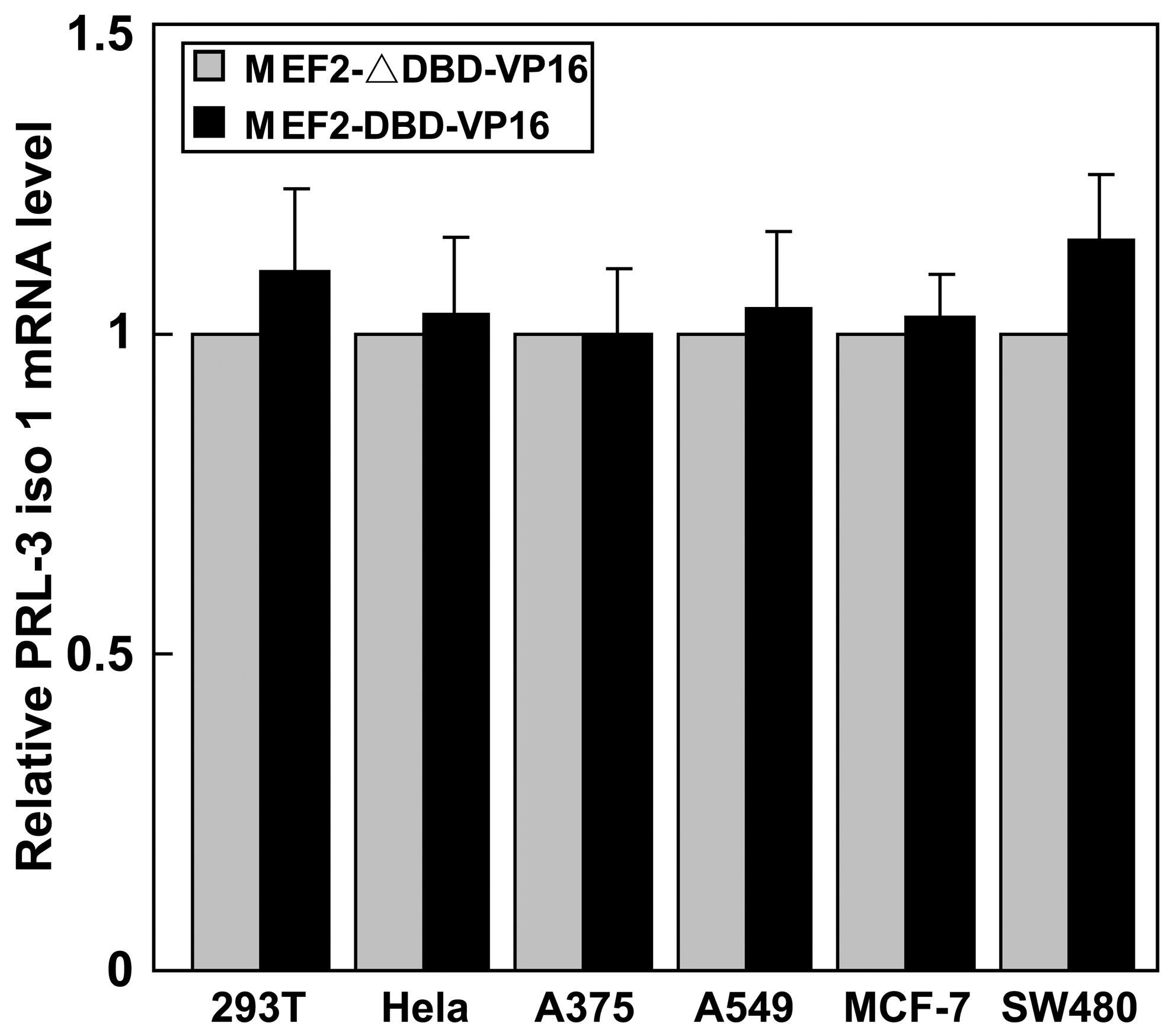

Supplement: Figure S3 — The constitutively active form of MEF2C has no effort on the abundance of the PRL-3-iso1 transcript. Cells were transfected with the plasmids indicated for 48 h and the abundance of the PRL-3-iso1 transcript was evaluated by real-time PCR. The expression data reflect mean ± SD from three independent experiments. (TIF) [file pone.0027165.s003.tif]

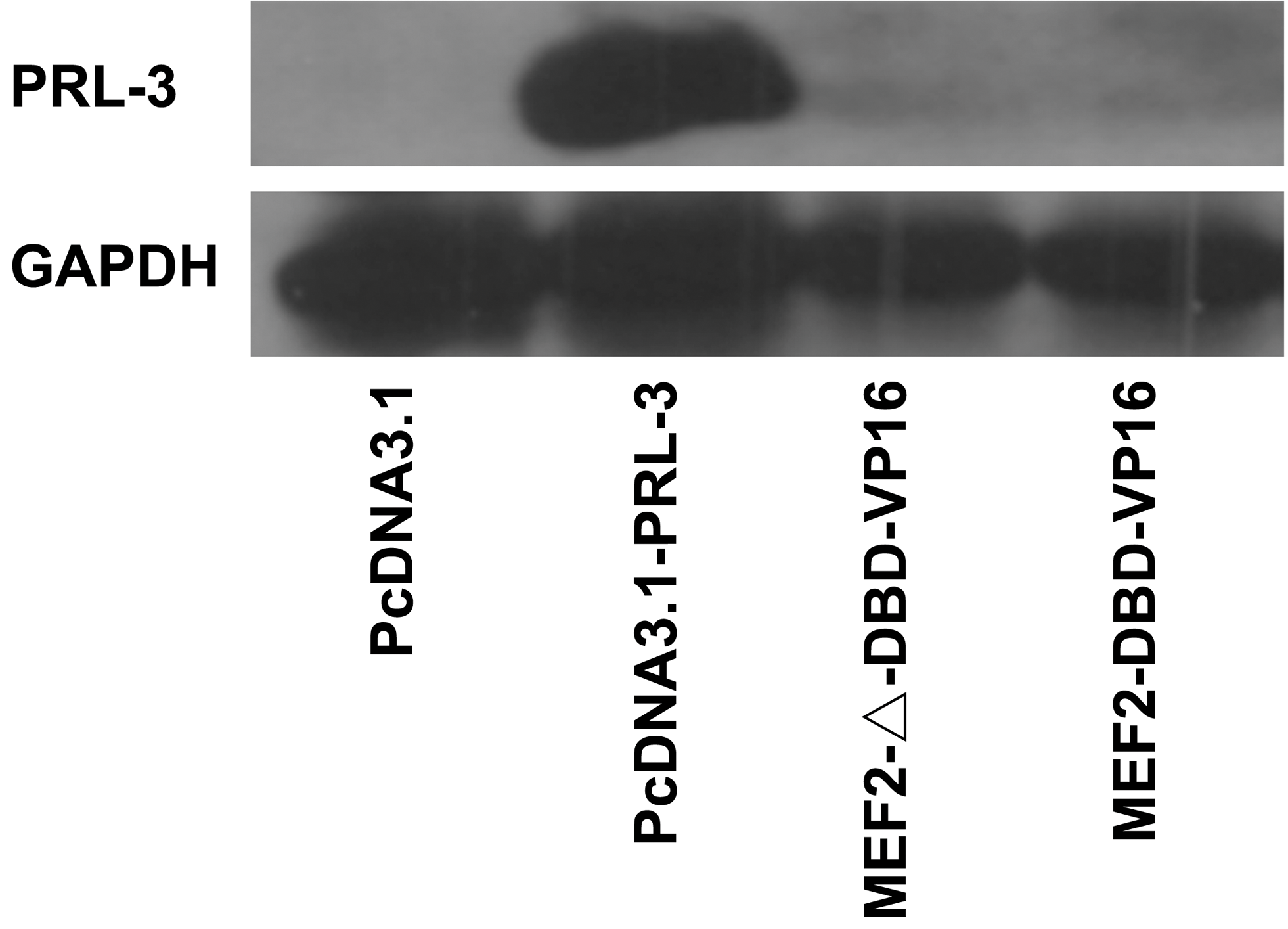

Supplement: Figure S4 — The constitutively active form of MEF2 has no effect on the abundance of the PRL-3 protein. The 293T cell line was transfected with the plasmids indicated for 48 h, and the abundance of PRL-3 and GAPDH was estimated by Western blots. Data shown are representative of three independent experiments. (TIF) [file pone.0027165.s004.tif]
