# Supplementary material for: VEGF Promotes the Transcription of the Human PRL-3 Gene in HUVEC through Transcription Factor MEF2C
Source: PLoS One. 2011 Nov 2;6(11):e27165. doi: 10.1371/journal.pone.0027165 (PMC3206935; doi:10.1371/journal.pone.0027165)
Supplement: Supplementary Sequence S1 — (DOC) [file pone.0027165.s005.doc]

**Supplementary Sequence: The two different *PRL-3* 5’UTR sequences identified by 5’RACE**

The underlined sequence is the first exon, according an alignment with DNA sequence of PRL-3. The first exon (76bp) of PRL-3-iso1 lies from 30232 to 30157bp upstream of the ATG start codon, and exon1 (92bp) of PRL-3-iso2 lies from 4313 to 4223bp upstream of the ATG start codon.

**PRL-3-iso1**

GCGGCCGCGCCTGTGCGCGAGGGCGCGCGCGTCCCGAGCCCTCCACCCGTCGTGCCGGCGCCGCCCGGACCGCCAGATGCTGTGTGCTGTGGACCCACCTGGGGTTCATGGAGTGGGCCACGGGGCCCAGCCCTAAGCACTGCTGCGCCCAGGGTCGCCGCGCCTCCTGCTGAGGGGTCCCCGTGCCACTGGCTCTCACCATTGCCCTCGCCTGCCAATGGCCTCTGCTGCCCAGCCTGGGGACAGCTCTACCGCCTGAGCCCCCTGCCCCACTCCAGGACTCACCGTACCCCGATGGGGTAACGTGACACAGGCCCCACACGTCAGAGGCCGCTGTCCCCACGGCCACTGCCCGTGACCCCTGGCCCAAGGCAGCTGGAGTTGGTTCAGTTCAAGTTCATTCTTCCTCTGGCCCTTGGGGGCTTGGGGCCCACCTCTGATTGAAGGGGGCTGTCTGCCCATCCACCAATGTGGAGAGGGCGCCCCCGGTGTGGGGTCCAGCTCTGGACACTGCTTGGCGGCCGGGTTCACTTTGAGTTTTTAAGTTTTCTTTGCTGAGCTTTTTTGGTTATTCTTTTTATTTTTTGCCTCTTTATGACTATCCAGCTCTGAGAGACGGGAGTTTGGAGTTGCCCGCTTTACTTTGGTTGGGTTGGGGGGGGCGGCGGGCTGTTTTGTTCCTTTTCTTTTTTAAGAGTTGGGTTTTCTTTTTTAATTATCCAAACAGTGGGCAGCTTCCTCCCCCACACCCAAGTATTTGCACAATATTTGTGCGGGGTATGGGGGTGGGTTTTTAAATCTCGTTTCTCTTGGACAAGCACAGGGATCTCGTTCTCCTCATTTTTTGGGGGTGTGTGGGGACTTCTCAGGTCGTGTCCCCAGCCTTCTCTGCAGTCCCTTCTGCCCTGCCGGGCCCGTCGGGAGGCGCC**ATG** (start codon of PRL-3)

**PRL-3-iso2**

GCGGCGGAGGGGCGGCGCCCCGACCCAGGCCCAGCACCGTGGGCACCGCCAGGGCCGGCGCGTATGGAGGCGGTGGGACGCCTGCGGCGCGGATGCTGTGTGCTGTGGACCCACCTGGGGTTCATGGAGTGGGCCACGGGGCCCAGCCCTAAGCACTGCTGCGCCCAGGGTCGCCGCGCCTCCTGCTGAGGGGTCCCCGTGCCACTGGCTCTCACCATTGCCCTCGCCTGCCAATGGCCTCTGCTGCCCAGCCTGGGGACAGCTCTACCGCCTGAGCCCCCTGCCCCACTCCAGGACTCACCGTACCCCGATGGGGTAACGTGACACAGGCCCCACACGTCAGAGGCCGCTGTCCCCACGGCCACTGCCCGTGACCCCTGGCCCAAGGCAGCTGGAGTTGGTTCAGTTCAAGTTCATTCTTCCTCTGGCCCTTGGGGGCTTGGGGCCCACCTCTGATTGAAGGGGGCTGTCTGCCCATCCACCAATGTGGAGAGGGCGCCCCCGGTGTGGGGTCCAGCTCTGGACACTGCTTGGCGGCCGGGTTCACTTTGAGTTTTTAAGTTTTCTTTGCTGAGCTTTTTTGGTTATTCTTTTTATTTTTTGCCTCTTTATGACTATCCAGCTCTGAGAGACGGGAGTTTGGAGTTGCCCGCTTTACTTTGGTTGGGTTGGGGGGGGCGGCGGGCTGTTTTGTTCCTTTTCTTTTTTAAGAGTTGGGTTTTCTTTTTTAATTATCCAAACAGTGGGCAGCTTCCTCCCCCACACCCAAGTATTTGCACAATATTTGTGCGGGGTATGGGGGTGGGTTTTTAAATCTCGTTTCTCTTGGACAAGCACAGGGATCTCGTTCTCCTCATTTTTTGGGGGTGTGTGGGGACTTCTCAGGTCGTGTCCCCAGCCTTCTCTGCAGTCCCTTCTGCCCTGCCGGGCCCGTCGGGAGGCGCC**ATG** (start codon of PRL-3)
