# Supplementary material for: VEGF Promotes the Transcription of the Human PRL-3 Gene in HUVEC through Transcription Factor MEF2C
Source: PLoS One. 2011 Nov 2;6(11):e27165. doi: 10.1371/journal.pone.0027165 (PMC3206935; doi:10.1371/journal.pone.0027165)
Supplement: Table S1 — Primers used for amplification of constructs of the PRL-3 promoter. (DOC) [file pone.0027165.s006.doc]

**Table S1.** Primers used for amplification of constructs of the *PRL-3 promoter*

| Constructs | Forward Primer (with *NheI* site) | Reverse Primer (with *HindIII* site) |
| --- | --- | --- |
| **P (-2131/+603)** | 5’-CGCGCTAGCGCATGGGGGGATGTGGCAG-3’ | 5’-CGCAAGCTTAGTCCATCCTTGTCCCTG-3’ |
| **P (-2131/+76)** | 5’-CGCGCTAGCAGCTCAGCATTTCCCCAG-3’ | 5’-CGCAAGCTTCCCACCGCCTCCATACGC-3’ |
| **P (-1740/+76)** | 5’-GCGGCTAGCGAGACCCCTTTTATGGATAAGAA-3’ | 5’-CGCAAGCTTCCCACCGCCTCCATACGC-3’ |
| **P (-1255/+76)** | 5’-CGCGCTAGCGGCTTGGTGTGCCCTTGGGTGGA-3’ | 5’-CGCAAGCTTCCCACCGCCTCCATACGC-3’ |
| **P (-826/+76)** | 5’-CGCGCTAGCGGAGGCAGAGCCAGGAGTCGCAG-3’ | 5’-CGCAAGCTTCCCACCGCCTCCATACGC-3’ |
| **P (-158/+76)** | 5’-CGCGCTAGCCGGCCCCTTTGTTTCCCGG-3’ | 5’-CGCAAGCTTCCCACCGCCTCCATACGC-3 |
| **P (-33/+76)** | 5’-CGCGCTAGCGCGGCGGTGGCGGCAGC-3’ | 5’-CGCAAGCTTCCCACCGCCTCCATACGC-3 |

*Note：the underlined nucleotides indicate Nhe*I *and Hind*III *recognition sites*
