# Supplementary material for: VEGF Promotes the Transcription of the Human PRL-3 Gene in HUVEC through Transcription Factor MEF2C
Source: PLoS One. 2011 Nov 2;6(11):e27165. doi: 10.1371/journal.pone.0027165 (PMC3206935; doi:10.1371/journal.pone.0027165)
Supplement: Table S2 — Primers used for Recombination PCR. (DOC) [file pone.0027165.s007.doc]

***Table S2.*** *Primers used for Recombination PCR*

| Primers | **MEF2-M1** | **MEF2-M2** |
| --- | --- | --- |
| **P1** | 5’-CGCGCTAGCCGGCCCCTTTGTTTCCCGG-3’ | 5’-CGCGCTAGCCGGCCCCTTTGTTTCCCGG-3’ |
| **P2** | 5’-CGCAGCCGCGCCCTGCCCGGGAAACAAAGGGGCCG-3’ | 5’-CGCCTGCGGCTGGCTATAGCGCCCGG-3’ |
| **P3** | 5’-CGGGCAGGGCGCGGCTGCGTTTAGGCGGCAGGTGTGG-3’ | 5’-CGCTATAGCCAGCCGCAGGCGGCGGTGGC-3’ |
| **P4** | 5’- CGCAAGCTTCCCACCGCCTCCATACGC -3’ | 5’- CGCAAGCTTCCCACCGCCTCCATACGC -3 |

*Note：the underlined nucleotides indicate Nhe*I *and Hind*III *recognition sites*
