# Supplementary material for: VEGF Promotes the Transcription of the Human PRL-3 Gene in HUVEC through Transcription Factor MEF2C
Source: PLoS One. 2011 Nov 2;6(11):e27165. doi: 10.1371/journal.pone.0027165 (PMC3206935; doi:10.1371/journal.pone.0027165)
Supplement: Table S3 — The sequences of MEF2 probes used for EMSA. (DOC) [file pone.0027165.s008.doc]

**Table S3.** The sequences of MEF2 probes used for EMSA

| Probes | **Sense** | **Antisense** |
| --- | --- | --- |
| **MEF2-M1-wt** | 5'-GGGCGCGGCTATATTTAGGCGGCAGG-3’ | 5'-CCTGCCGCCTAAATATAGCCGCGCCC-3’ |
| **MEF2-M1-mut** | 5'-GGGCGCGGCTATAGCCAGGCGGCAGG-3’ | 5'-CCTGCCGCCTGGCTATAGCCGCGCCC-3’ |
| **MEF2-M2-wt** | 5'- CCCGGGCGCTATAAATAGCCGCAGGC-3’ | 5'-GCCTGCGGCTATTTATAGCGCCCGGG-3’ |
| **MEF2-M2-mut** | 5'-CCCGGGCGCTATAGCCAGCCGCAGGC-3’ | 5'-GCCTGCGGCTGGCTATAGCGCCCGGG-3’ |

*Note：the underlined nucleotides indicate sites of mutation*
