# Supplementary material for: VEGF Promotes the Transcription of the Human PRL-3 Gene in HUVEC through Transcription Factor MEF2C
Source: PLoS One. 2011 Nov 2;6(11):e27165. doi: 10.1371/journal.pone.0027165 (PMC3206935; doi:10.1371/journal.pone.0027165)
Supplement: Table S4 — Primers used for RT-PCR and Real-time PCR. (DOC) [file pone.0027165.s009.doc]

***Table S4.*** *Primers used for RT-PCR and Real-time PCR*

| Primers | **Sense** | **Antisense** |
| --- | --- | --- |
| **hPRL3** | 5’-GGGACTTCTCAGGTCGTGTC-3’ | 5’-AGCCCCGTACTTCTTCAGGT-3’ |
| **hMEF2C** | 5’-TTCCAGTATGCCAGCACCG-3’ | 5’-GGCCCTTCTTTCTCAACGTCTC-3’ |
| **β-Actin** | 5’-CGACAGGATGCAGAAGGAGA-3’ | 5’-CGTCATACTCCTGCTTGCTG-3’ |
| **PRL-3-iso1** | 5’-GAGCCCTCCACCCGTCGTGC-3’ | 5’-ATCGGCAGGCGAGGGCAATGG-3’ |
| **PRL-3-iso2** | 5’-GCGTATGGAGGCGGTGGGAC-3’ | 5’-ATCGGCAGGCGAGGGCAATGG-3’ |
