# Supplementary material for: VEGF Promotes the Transcription of the Human PRL-3 Gene in HUVEC through Transcription Factor MEF2C
Source: PLoS One. 2011 Nov 2;6(11):e27165. doi: 10.1371/journal.pone.0027165 (PMC3206935; doi:10.1371/journal.pone.0027165)
Supplement: Table S5 — siRNA sequences used for MEF2C and PRL-3 knockdown experiments. (DOC) [file pone.0027165.s010.doc]

***Table S5.*** *siRNA sequences used for MEF2C and PRL-3 knockdown experiments*

|  | **Sense** | **Antisense** |
| --- | --- | --- |
| **MEF2C #1** | 5’-GACCUGUCAUCUCUGUCUGGGUUUA-3’ | 5’- UAAACCCAGACAGAGAUGACAGGUC -3’ |
| **MEF2C #2** | 5’- GGAGCUUGCACUAGCACUCAUUUAU-3’ | 5’- AUAAAUGAGUGCUAGUGCAAGCUCC -3’ |
| **MEF2C #3** | 5’-CAGCGCUCUUCACCUUGGUUCAGUA-3’ | 5’- UACUGAACCAAGGUGAAGAGCGCUG -3’ |
| **PRL-3** | 5’- UCACCUACCUGGAGAAAUA-3’ | 5’- UAUUUCUCCAGGUAGGUGA -3’ |
